# Anti-inflammatory and antiarthritic effects of piperine in human interleukin 1β-stimulated fibroblast-like synoviocytes and in rat arthritis models

**DOI:** 10.1186/ar2662

**Published:** 2009-03-30

**Authors:** Jun Soo Bang, Da Hee Oh, Hyun Mi Choi, Bong-Jun Sur, Sung-Jig Lim, Jung Yeon Kim, Hyung-In Yang, Myung Chul Yoo, Dae-Hyun Hahm, Kyoung Soo Kim

**Affiliations:** 1 East-West Bone & Joint Research Institute, East-West Neo Medical Center, Kyung Hee University, 149 Sangil-dong, Gangdong-gu, Seoul, Republic of Korea; 2 Acupuncture and Meridian Science Research Center, Kyung Hee University, Hoeggidong, Dongdaemoon-gu, Seoul, Republic of Korea; 3 Department of Pathology, East-West Neo Meidcal Center, Kyung Hee University, 149 Sangil-dong, Gangdong-gu, Seoul, Republic of Korea; 4 Department of Pathology, Inje University Sanggye Paik Hospital, Sanggye 7 dong 761-7, Nowon-gu, Seoul, Republic of Korea; 5 Department of Internal Medicine, East-West Neo Medical Center, Kyung Hee University, 149 Sangil-dong, Gangdong-gu, Seoul, Republic of Korea; 6 Department of Orthopedic Surgery, East-West Neo Medical Center, Kyung Hee University, 149 Sangil-dong, Gangdong-gu, Seoul, Republic of Korea

## Abstract

**Introduction:**

The objective of this study was to determine the anti-inflammatory, nociceptive, and antiarthritic effects of piperine, the active phenolic component in black pepper extract.

**Methods:**

The *in vitro *anti-inflammatory activity of piperine was tested on interleukin 1β (IL1β)-stimulated fibroblast-like synoviocytes derived form patients with rheumatoid arthritis. The levels of IL6, matrix metalloproteinase (MMPs), cyclo-oxygenase 2 (COX-2), and prostaglandin E2 (PGE_2_) were investigated by ELISA and RT-PCR analysis. The analgesic and antiarthritic activities of piperine were investigated on rat models of carrageenan-induced acute paw pain and arthritis. The former were evaluated with a paw pressure test, and the latter by measuring the squeaking score, paw volume, and weight distribution ratio. Piperine was administrated orally to rats at 20 and 100 mg/kg/day for 8 days.

**Results:**

Piperine inhibited the expression of IL6 and MMP13 and reduced the production of PGE_2 _in a dose dependant manner at concentrations of 10 to 100 μg/ml. In particular, the production of PGE_2 _was significantly inhibited even at 10 μg/ml of piperine. Piperine inhibited the migration of activator protein 1 (AP-1), but not nuclear factor (NF)κB, into the nucleus in IL1β-treated synoviocytes. In rats, piperine significantly reduced nociceptive and arthritic symptoms at days 8 and 4, respectively. Histological staining showed that piperine significantly reduced the inflammatory area in the ankle joints.

**Conclusions:**

These results suggest that piperine has anti-inflammatory, antinociceptive, and antiarthritic effects in an arthritis animal model. Thus, piperine should be further studied with regard to use either as a pharmaceutical or as a dietary supplement for the treatment of arthritis.

## Introduction

Rheumatoid arthritis is characterized by chronic proliferative synovitis, inflammatory immune cell infiltration into the synovial fluid and cartilage destruction [1]. Proliferative fibroblast-like synoviocytes (FLSs) play crucial roles in both the propagation of inflammation and joint damage because they produce a great amount of proinflammatory mediators such as matrix metalloproteinses (MMPs), interleukin (IL)6, IL8 and prostaglandin E_2 _(PGE_2_) [2].

Thus, anti-inflammatory agents are administrated as long-term treatments for patients with rheumatoid arthritis. However, anti-inflammatory agents carry the risk of gastrointestinal toxicity; thus, their use is limited. In an attempt to avoid adverse gastrointestinal effects, a new generation of non-steroidal anti-inflammatory drugs (NSAIDs) was developed that selectively inhibited cyclo-oxygenase (COX)-2 selective inhibitors (for example, celecoxib, rofecoxib, and valdecoxib) [3]. Celecoxib and valdecoxib appear to show satisfactory cardiovascular safety, however, rofecoxib was withdrawn from the market due to cardiovascular toxicity. However, side effects remain one of the problems for long-term use; thus, there is a need for anti-inflammatory drugs with less severe side effects. In addition, recent interest in alternative treatments for arthritis [4,5] has promoted their use in the US, but scientific evidence of antiarthritic efficacy is lacking.

Black pepper (*Piper nigrum*) is commonly used as a spice in human diets, but it is also used as a medicine, a preservative, and a perfume in many Asian countries. An extract of the active phenolic component, piperine, is well known to provide beneficial physiological effects [6]. It stimulates the digestive enzymes of pancreas, protects against oxidative damage, lowers lipid peroxidation, and enhances the bioavailability of a number of therapeutic drugs. In addition, its anti-inflammatory activities have been demonstrated in rat models of carrageenan-induced rat paw edema, cotton pellet-induced granuloma, and a croton oil-induced granuloma pouch [7]. Constituents of the piper species have shown *in vitro *inhibitory activity against the enzymes responsible for leukotriene and prostaglandin biosynthesis, 5-lipoxygenase and COX-1, respectively [8]. These effects of piperine seem to be beneficial for inflammatory diseases that are accompanied by severe pain; for example, rheumatoid arthritis.

The excellent therapeutic properties of piperine have been demonstrated in various cell types [9-12]. Nevertheless, little is known about the effect of piperine on the production of proinflammatory mediators in FLSs. Furthermore, to our knowledge, its antiarthritic efficacy has never been evaluated. In this study, the anti-inflammatory effects of piperine were tested in IL1β-stimulated rheumatoid arthritis fibroblast-like synoviocytes derived from patients with rheumatoid arthritis. Its antiarthritic efficacy was evaluated in animal models of experimental arthritis.

## Materials and methods

### Cell culture and reagents

All *in vitro *experiments were carried out with fibroblast-like synoviocytes derived from patients with rheumatoid arthritis (RA). After obtaining informed consent, synovial tissues were collected from RA patients. They met the 1987 American College of Rheumatology (ACR) criteria for the diagnosis of RA and had been treated with non-biological disease-modifying antirheumatic drugs (DMARDs) and were underwent therapeutic joint surgery. FLSs were isolated as described previously [13] and grown in Dulbecco's Modified Eagle Medium (DMEM, low glucose) (Gibco-BRL, Grand Island, NY, USA) supplemented with 10% (v/v) fetal bovine serum (FBS; Gibco-BRL) and 1 × antibiotic-antimycotic (Gibco-BRL). After the cells had grown to confluence, they were split at a 1:4 ratio. FLS passages 3 to 6 from three patients were used for all experiments. Piperine, prednisolone, corn oil and carrageenan were obtained from Sigma-Aldrich Korea (Young-In, Korea). Celecoxib was purchased in the form of the commercial drug, Celebrex (capsules; Pfizer Korea, Seoul, Korea).

### Semiquantitative RT-PCR

FLSs (2.5 × 10^5 ^cells) were cultured overnight in 60 mm dishes containing 2 ml of media. Cells were incubated with serum-free media for 2 h and new serum-free media was replaced just prior to the addition of piperine and cultured for 24 h. Supernatants were collected for ELISA and the cells were used for semiquantitative RT-PCR. Trizol was used to extract total RNA from the cells. Complementary DNA was synthesized from 1 μg of total RNA in a 20 μl reverse transcription reaction mixture. For semiquantitative PCR, aliquots of cDNA were amplified in a 25 μl PCR mixture according to the protocol provided by the manufacturer (TaKaRa Bio, Kyoto, Japan), as described previously [13]. The PCR conditions for the MMPs, IL6, and COX-2 were as follows: 30 to 33 cycles of 95°C for 45 s, 55 to 60°C for 45 s, and 72°C for 45 s. PCR products were subjected to electrophoresis on 1.5% agarose gels containing ethidium bromide, and the bands were visualized under ultraviolet (UV) light.

### ELISA

Synovial cells (2.5 × 10^5 ^cells/60 mm dish/2 ml serum-free media) were treated with various concentrations of piperine 30 minutes prior to IL1β stimulation. Conditioned media was collected 24 h later. Briefly, FLS cultures were centrifuged and the supernatants were collected and analyzed for IL6, PGE_2_, MMP1, and MMP13 with an ELISA kit (R&D Systems, Minneapolis, MN, USA). For mRNA analysis, the cells were lysed and total RNA was extracted. The mRNA levels of IL6 and COX-2 were measured by semiquantitative RT-PCR analysis. The COX-2 protein expression was measured by western blot. Three independent experiments were performed in duplicate. Each experiment was performed using synovial cells from different patients. The collected supernatants were analyzed for IL6, PGE_2_, MMP1 and MMP13 using commercial kits (ELISA; R&D Systems). For the measurement of transcription factors, nuclear factor (NF)κB and activator protein 1 (AP-1), in the nucleus, FLSs were seeded (5 × 10^6 ^cells) into 100 mm dishes and grown to 80% confluence. The cells were serum-starved overnight and stimulated by IL1β (10 ng/ml) for 90 minutes in the presence or absence of piperine. Subsequently, the cells were washed twice in phosphate-buffered saline (PBS) and treated with lysis buffer and the extraction of transcription factors from the nucleus was performed according to the manufacturer's protocol (Active Motif, Seoul, Korea).

### Western blot analysis

FLSs cultured (2.5 × 10^5 ^cells) in 60 mm dishes were serum-starved overnight and stimulated by IL1β (10 ng/ml) for 10 or 30 minutes in the presence or absence of piperine. The cells were subsequently washed twice in PBS and treated with 50 μl of lysis buffer (20 mM Tris-Cl pH 8.0, 150 mM NaCl, 1 mM ethylenediaminetetraacetic acid (EDTA), 1% Triton X-100, 20 μg/ml chymostatin, 2 mM phenylmethylsulfonyl fluoride (PMSF), 10 μM leupeptin, and 1 mM 4-(2-aminoethyl)benzenesulfonyl fluoride (AEBSF)). As described previously [13], the samples were separated using 12% SDS-PAGE, and were then transferred to Hybond-ECL membranes (Amersham, Arlington Heights, IL, USA). The membranes were first blocked with 6% non-fat milk dissolved in Tris-buffered saline/Tween (TBST) buffer (10 mM Tris-Cl pH 8.0, 150 mM NaCl, 0.05% Tween 20). The blots were then probed with various rabbit polyclonal antibodies for inhibitor of κB (IκB)α, p-ERK1/2, p-P38, p-Jun N-terminal kinase (JNK) and β-actin (Cell Signaling Technology, Beverly, MA, USA) diluted 1:1,000 in TBS at 4°C for overnight, and incubated with 1:1,000 dilutions of goat anti-rabbit IgG secondary antibody coupled with horseradish peroxidase. The blots were developed using the ECL method (Amersham). For re-probing, the blots were incubated in the stripping buffer (100 mM 2-mercaptoethanol, 2% SDS, 62.5 mM Tris-HCl pH 6.7) at 50°C for 30 minutes with occasional agitation.

### Histological assessment of inflammation

The rats were killed after 9 days of carrageenan and control treatments. Immunohistochemical staining was performed to determine the degree of immune cells infiltration into the joints. Rat ankles were dissected, fixed in 10% formalin, dehydrated through a graded ethanol series, cleared in xylene, and processed for embedding in paraffin wax with routine protocols. A microtome was used to cut 4 μm-thick sections that were subsequently stained with hematoxylin and eosin (H&E) stain. The degree of inflammation was evaluated on a scale from 0 to 5 by three different pathologists that had been blinded to the treatments. The scale was defined as follows: 0 = no inflammation, 1 = mild inflammation, 2 = mild/moderate inflammation, 3 = moderate inflammation, 4 = moderate/severe inflammation, and 5 = severe inflammation.

### Rat models of paw hyperalgesia and arthritis

Sprague-Dawley 5-week-old to 6-week-old male rats, purchased from SLC (Shizuoka, Japan), were used in this study. All animals were maintained in plastic cages at 21 to 24°C under a 12 h light/dark cycle and were given free access to pellet food and water. They were adapted for at least 1 week prior to the start of the experiment. All subjects were habituated to the behavioral test chambers and handled with special care to minimize stress. All methods were approved by the Animal Care and Use Committee of Kyung Hee University. All procedures were conducted in accordance with the *Guide for the Care and Use of Laboratory Animals*, published by the Korean National Institute of Health.

To induce paw hyperalgesia, rats were given an intraplantar injection of 1% carrageenan (0.1 ml) in the posterior right paw as described previously [14]. After 3 hr of the injection, the pain threshold was measured using a paw pressure analgesia instrument (UGO-BASIL Biological Research Apparatus, Comerio-Varese, Italy) for the Randall-Selitto test paw. A total of 10 rats were studied per group and the test was performed blind. Rats were starved overnight and piperine was evaluated at doses of 20 and 100 mg/kg. Piperine dissolved in corn oil was fed orally 1 h before carrageenan injection. To evaluate paw hyperalgesia, we measured the tolerance to increasing mild pressure on the affected paw between a flat surface and a blunt pointer of the instrument, as manufacturer's protocols. The effects of piperine were compared to the effects of Celebrex (Pfizer), a selective COX-2 inhibitor (100 mg/kg).

The carrageenan-induced arthritic rat model was prepared as described previously [15]. Animals were briefly anesthetized with 3% isoflurane in a mixed N_2_O/O_2 _gas. Arthritic inflammation was induced by a single injection of 3% carrageenan suspended in 100 μl of pyrogen-free sterile saline, into the left tibiotarsal ankle joint. The effects of piperine were compared to the effects of prednisolone (10 mg/kg), a corticosteroid.

To evaluate the arthritic progression of carrageenan-induced arthritis in the rat, three different parameters were measured: paw volume, squeaking score in the ankle flexion test, and weight distribution ratio (WDR). These were considered behavioral indicators of carrageenan-induced arthritis and checked daily for 9 days. With progression of arthritis, redness and swelling of the ankle joints and arthritic pain started to appear and reached a maximum on day 1 after the carrageenan injection. At that time, piperine and prednisolone dissolved in corn oil was administrated orally once a day for 8 days.

The paw volumes were measured using a digital plethysmometer (UGO-BASIL Biological Research Apparatus), as described by Kwon *et al. *[16]. Paw volumes were expressed as relative values to that of day 0 when carrageenen was injected. The ankle flexion test involved gentle flexion and extension of the carrageenan-injected ipsilateral hind limb, as described by Kwon *et al. *[16]. This elicited vocalizations (squeaking) that were scored on a scale (squeaking score) as a measure of hyperalgesia. The procedure of flexion and extension were repeated 10 times in every 5 s and the rating of 0 (null) or 1 (vocalization) was given to each hind limb. This test was performed only once a day in each animal. The WDR is a ratio of the percentage of weight carried on each hind leg in which the weight-bearing forces of both hind limbs were measured with an incapacitance meter (UGO-BASIL Biological Research Apparatus), as previously described by Hwang *et al*. [15]. To evaluate arthritic pain, the rat was placed in the test box of an incapacitance meter in which a slanted plank is located. The bearing force of each hind limb was quantified by two mechanotransducers, separately placed below the two hind limbs: one is normal and the other is the arthritic limb. The bearing force of each hind limb was estimated as a 5-s average, and the mean bearing force was calculated from four separate estimations. The WDR percentage was calculated as percentage WDR = 100 × (weight borne by ipsilateral limb/total weight borne by both limbs). The WDR of the hind paws in the normal group was 50:50 (data not shown), indicating that 50% of the weight was carried in each hind paw. As the pain and swelling of the ankle progressed due to induction of arthritis, the balance of weight was disrupted, resulting in a reduction of the WDR in the arthritic leg. All behavioral tests were performed blinded.

### Statistical analysis

The *in vitro *experimental data are expressed as the mean ± standard error of the mean (SEM) of three independent experiments. The *in vivo *experimental data are presented as the mean ± SEM. The differences between groups were assessed by repeated analysis of variance (ANOVA), followed by the Tukey "honestly significantly different" (HSD) *post hoc *analysis. The degree of inflammation observed in H&E stained sections was compared between groups with the Mann-Whitney test. Differences were considered significant at *P *< 0.05.

## Results

### Effect of piperine on FLS production of inflammatory mediators

**Figure 1:**
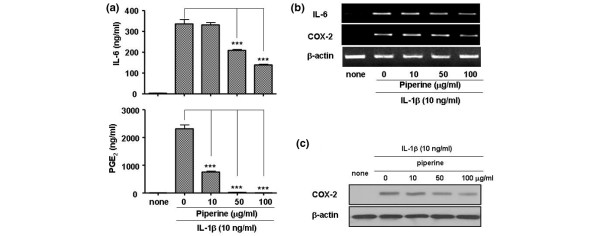
Effect of piperine on the production of proinflammatory mediators. **(a) **ELISA results show that piperine inhibited the production of interleukin (IL)6 and prostaglandin E_2 _(PGE_2_) in IL1β-stimulated fibroblast-like synoviocytes (FLSs) in a dose-dependent manner. **(b) **Piperine effects on IL6 and cyclo-oxygenase (COX)-2 mRNA expression measured by semiquantitative RT-PCR. **(c) **Piperine effects on COX-2 protein expression measured by western blot. Experiments were performed with synovial cells derived from patients with rheumatoid arthritis. Values are expressed ± standard error of the mea (SEM). ***P < 0.001 vs IL1β treated group without piperine.

To test the anti-inflammatory efficacy of piperine, FLSs were stimulated with IL1β at 10 ng/ml in the presence or absence of piperine. The addition of IL1β significantly increased the production of IL6 and PGE_2 _compared to that of controls (no IL1β). The addition of piperine greatly inhibited the IL6 and PGE_2 _response to IL1β in dose-dependent manner (Figure 1). Piperine also inhibited both the protein and mRNA expression levels of IL6 and COX-2. In particular, piperine inhibited the production of PGE_2 _more potently than the production of IL6. Interestingly, piperine inhibited the expression of the COX-2 protein more significantly than the COX-2 mRNA.

**Figure 2:**
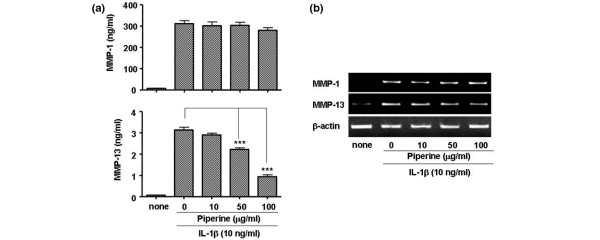
Effect of piperine on the production of extracelluar matrix degradation enzymes (matrix metalloproteinases (MMPs)). **(a) **ELISA results show that piperine inhibited the production of MMP13, but not MMP1 proteins, in interleukin (IL)1β-stimulated fibroblast-like synoviocytes (FLSs) in a dose dependent manner. **(b) **mRNA levels were measured by semiquantitative RT-PCR. Experiments were performed with synovial cells derived from patients with rheumatoid arthritis. Values are expressed ± standard error of the mean (SEM). ***P < 0.001 vs IL1β treated group without piperine.

**Figure 3:**
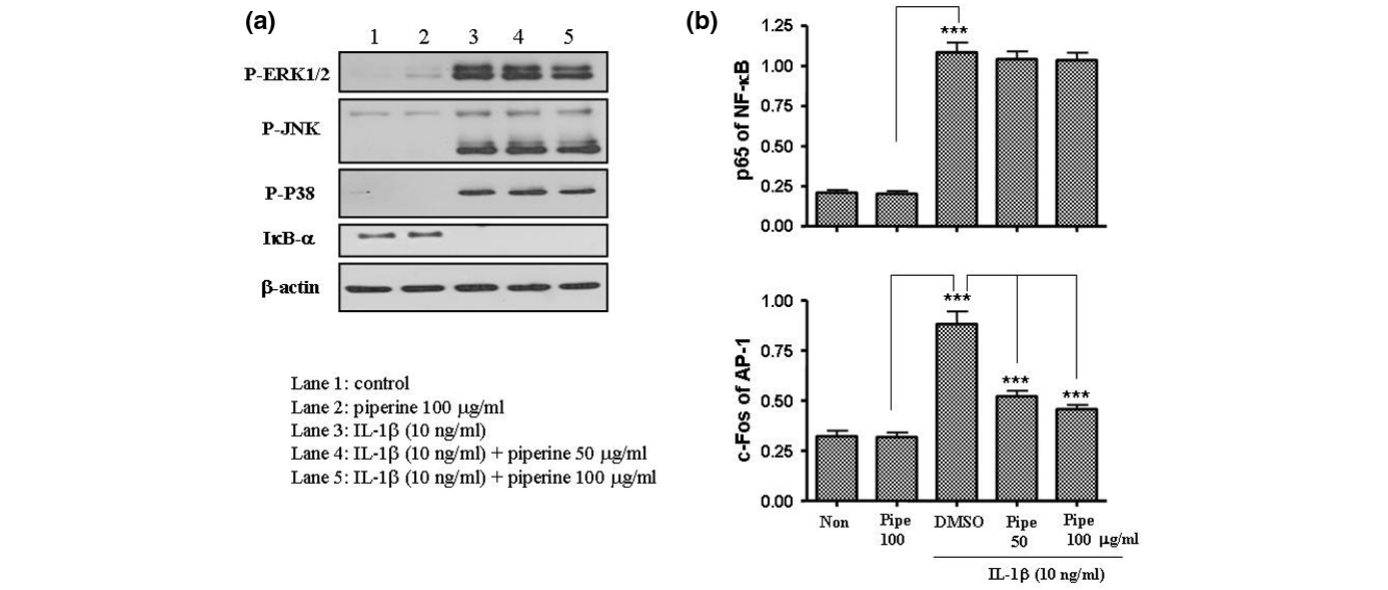
Effects of piperine on signaling pathways and transnuclear migration. **(a) **Interleukin (IL)1β-stimulated fibroblast-like synoviocytes (FLSs) treated with piperine were analyzed by western blot. Piperine treatment did not inhibit the degradation of inhibitor of κB (IκB)α, but slightly inhibited the phosphorylation of extracellular-regulated kinase (ERK)1/2 in the MAP kinase signaling pathways was slightly inhibited in the presence of piperine. **(b) **The nuclear levels of nuclear factor (NF)κB and activator protein 1 (AP-1) were measured by ELISA detecting p65 and c-FOS from nuclear extracts, respectively, on an ELISA. Piperine inhibited the level of AP-1 in the nucleus, but not NFκB levels. Values are expressed ± standard error of the mean (SEM). ***P < 0.001 vs IL1β treated group without piperine or piperine alone.

Next, we tested whether piperine inhibited the expression of the extracellular matrix degradation enzymes (MMPs). MMP1 and MMP13 play an important role in degrading cartilage in IL1β-stimulated FLSs. We found that piperine inhibited MMP13 expression at both the protein and mRNA levels, but not MMP1 (Figure 2). To understand the molecular mechanisms underlying piperine inhibition of IL6, COX-2 and MMP expression, we investigated the MAP kinase and IκB kinase signaling pathways by western blot (Figure 3a). Interestingly, piperine did not significantly affect the IκB kinase signaling pathway or the MAP kinase mediated phosphorylation of JNK and P38; however, piperine slightly inhibited the MAP kinase mediated phosphorylation of ERK1/2. In addition, piperine reduced the level of AP-1 that migrated to the nucleus (in response to IL1β) in a dose dependent manner, but did affect the levels of NFκB in nucleus (Figure 3b). This suggested that piperine inhibition of the ERK1/2 signaling pathway blocked the migration of AP-1 into the nucleus.

### Analgesic effect of piperine in carrageenan-induced paw hyperalgesia

**Figure 4:**
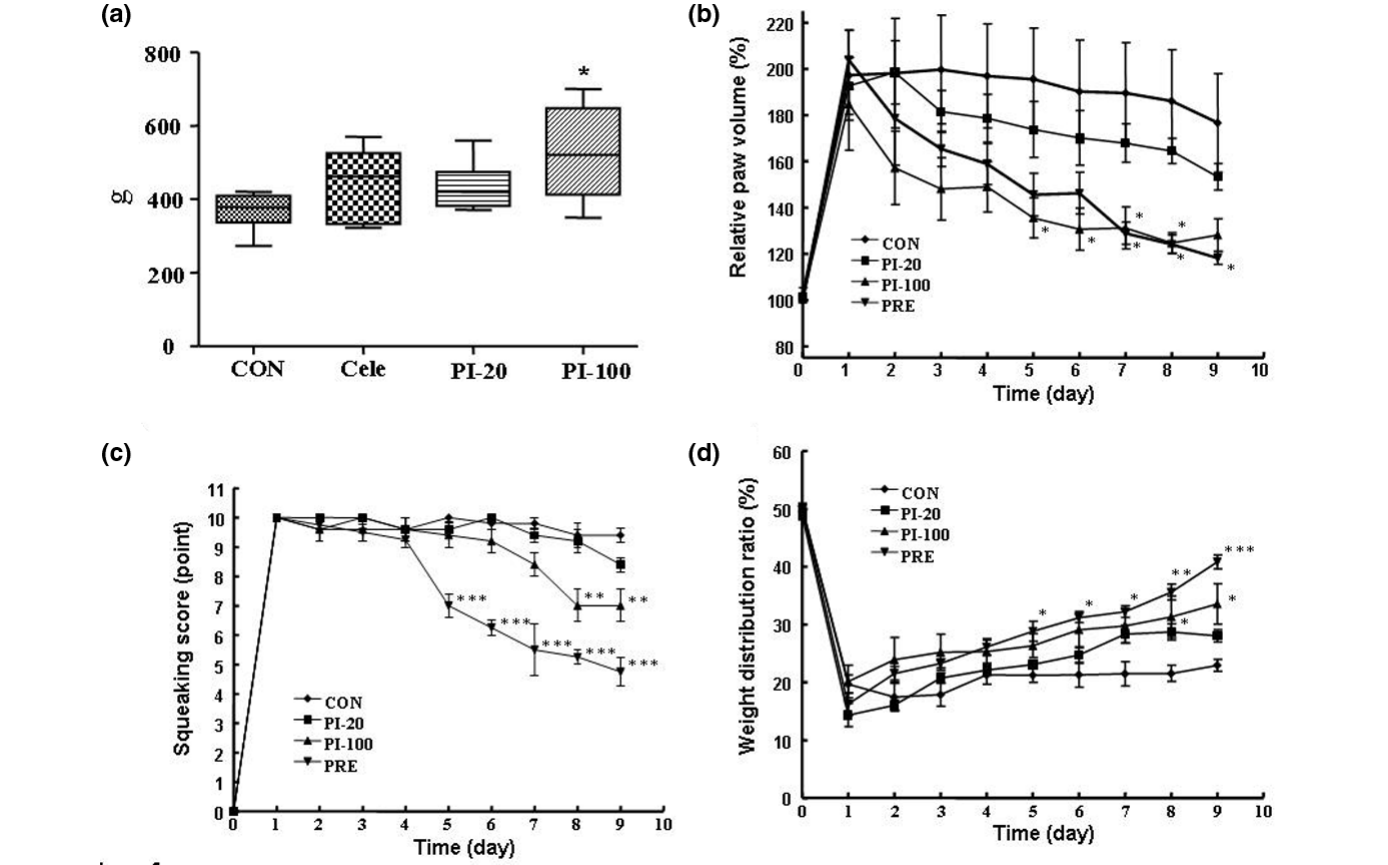
Analgesic and antiarthritic effects of piperine in rat models of paw edema and arthritic ankle. **(a) **Piperine showed analgesic effects in carrageenan-induced paw edema. The y axis indicated the pressure (g) that was tolerated before the rat exhibited signs of pain. Arthritic symptoms were measured by **(b) **relative paw volume, expressed as a function of the unaffected paw (100%). **(c) **The ankle flexion pain score (a value of 0 represents no indication of pain); and **(d) **the weight distribution ratio (a value of 50% indicated that weight was equally distributed between the two hind paws). The results indicated that piperine had antiarthritic effects. Con = control mice, Cele = celecoxib (100 mg/kg), PI-20/PI-100 = pierine at 20/100 mg/kg, Pre = prednisolne. Values are expressed ± standard error of the mean (SEM). *P < 0.05, **P < 0.01, ***P < 0.001 vs control group.

Because piperine significantly inhibited the production of PGE_2 _and the protein levels of COX-2, we tested whether piperine had antinociceptive effects in a rat model of carrageenan-induced paw hyperalgesia. We found in paw pressure tests that rats treated with piperine could tolerate higher pressures on the affected paw (Figure 4a). The efficacy at 100 mg/kg was better than that of celecoxib, and at a dose of 20 mg/kg, piperine showed a mild analgesic effect.

### Antiarthritic effect of piperine on the carrageenan-induced arthritis rat model

To demonstrate the *in vivo *antiarthritic effect of piperine, the efficacy of piperine was tested in a rat model of carrageenan-induced arthritis. The piperine (100 mg/kg) group showed a significant reduction in paw volume compared to the vehicle-treated arthritic group (Figure 4). At this dose, piperine showed almost the same efficacy as prednisolone (10 mg/kg), which was used as a positive control. Piperine also provided a mild antiedema effect at 20 mg/kg, although it was not statistically significant.

The vocalizations caused by flexion or extension of the inflamed ankle reached a maximum point on day 1 after the carrageenan injection and was sustained at a maximum level in untreated rats through the end of the experiment (Figure 4c). In the100 mg/kg piperine treated group, the number of vocalizations started to decrease at 5 days post-carrageenan injection. At 20 mg/kg, piperine exhibited little analgesic effect. Next, we measured the analgesic effect of piperine (20 and 100 mg/kg) on the weight distributed on the hind paws (WDR) of rats with carrageenan-induced arthritis in one paw (Figure 4d). Before the carrageenan injection (day 0), the mean WDR did not differ significantly among the experimental groups (the WDR was 50:50, thus controls carried 50% of the weight on each hind paw). However, significant changes in the ratio were observed on day 1 after the carrageenan injection, and the weight carried by the affected leg in the vehicle-treated arthritic group (CON) reached 20% at day 9. Distinct recovery of WDR was observed in groups that received 20 and 100 mg/kg piperine on days 8 and 9, despite the statistically insignificant analgesic effect of 20 mg/kg piperine.

### Anti-inflammatory effect of piperine by histological evaluation

**Figure 5:**
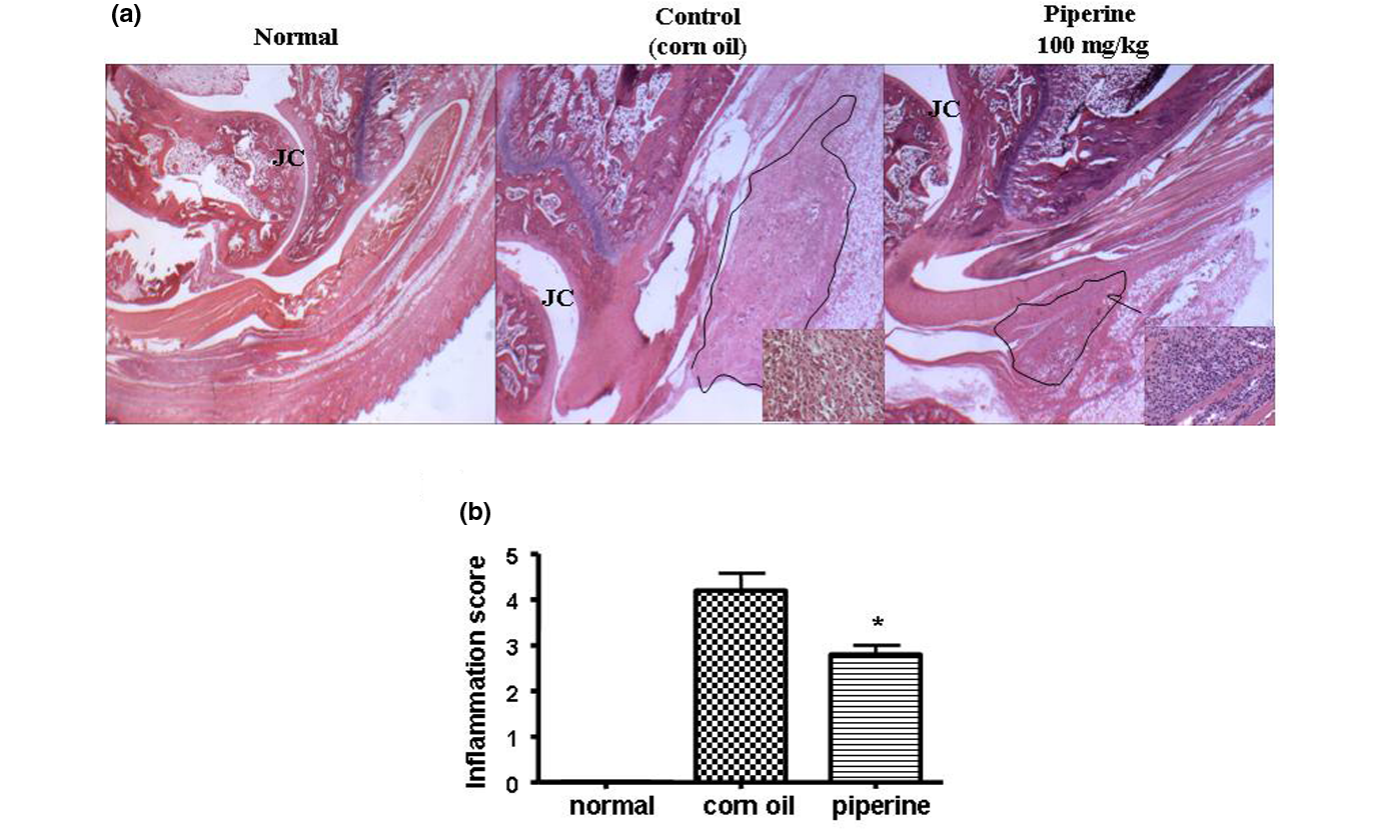
Histological evaluation of the anti-inflammatory effects of piperine. Paraffin sections of rat ankles were stained with hematoxylin and eosin (H&E). **(a) **Histopathological analysis showed that piperine (100 mg/kg) significantly inhibited ankle inflammation. Each photo is representative of five specimens for each group (original magnification × 100). The insets are enlargements of the regions outlined in black, and show the infiltrates at a magnification of × 200. **(b) **The degree of inflammation was evaluated on a scale from 0 to 5 by three pathologists that were blinded to the treatments. Values are expressed ± standard error of the mean (SEM). *P < 0.05 vs corn oil treated group.

To evaluate the anti-inflammatory effects of piperine, samples of the ankle joints from each experimental group were examined by H&E staining. We found that the group that received piperine (100 mg/kg) had significantly smaller areas of lymphocyte infiltration into the joints compared to the corn oil treated group (Figure 5). The degree of inflammation in five specimens was evaluated by three different pathologists. The scores indicated that piperine significantly reduced the inflammation induced by carrageenan (Figure 5b).

## Discussion

Anti-inflammatory drugs used for treating chronic inflammatory diseases such as rheumatoid arthritis are typically prescribed long term to properly control the disordered immune system. Thus, there is a strong need to develop safe and effective drugs for the long-term use. Many groups have studied non-steroidal anti-inflammatory small molecules that were derived from natural sources with the aim of developing new treatments for clinical use [17]. For example, curcumin is a polyphenolic compound derived from the dietary spice, turmeric. Recently, curcumin has been shown to possess diverse pharmacological properties, including anti-inflammation, antiproliferation, and antiangiogenesis. Currently, curcumin is in phase I of clinical trials [18].

Piperine is also a promising natural source with potential for clinical use. *Piper longum *Linn. has been used in Asia as a natural treatment for poor peripheral blood circulation [19]. *Piper longum *Linn. and *Piper nigrum *Linn. are conventionally used as immune enhancers in Indian traditional medicine [20]. Therefore, piperine has been proven effective indirectly, but its mechanism of action remains unknown. In the present study, we evaluated the anti-inflammatory and antiarthritic effects of piperine to determine whether it had therapeutic potential for the treatment of arthritis.

We found that piperine significantly inhibited the production of two important proinflammatory mediators, IL6 and PGE_2_, in IL1β-stimulated human FLS. This result was consistent with other studies that showed potent anti-inflammatory effects in other systems. The inhibition of PGE_2 _production is important due to its central role in triggering pain. In addition, MMP1 and MMP13 collagenases play dominant roles in RA and osteoarthritis because they are the rate-limiting components of the collagen degradation process. The significant inhibition of MMP13 expression is particularly important because it degrades a wide range of collagenous and non-collagenous extracellular matrix macromolecules and is remarkably active against collagen type II, the predominant collagen in cartilage. To our knowledge, this is the first report to show that piperine inhibited the expression of MMP13 in IL1β-stimulated FLSs.

We also investigated the molecular mechanisms underlying piperine inhibition. We found that piperine did not significantly inhibit the activation of MAP kinase or IκB kinase signaling pathways. At the maximum concentration tested (100 μg/ml), piperine slightly inhibited the phosphorylation of ERK1/2 stimulated by IL1β. Piperine also inhibited the activation of the transcription factor AP-1, but not NFκB, in our system. However, a previous study in B16F-10 melanoma cells showed that piperine was able to inhibit the activation of several transcription factors, including NFκB, c-FOS, cAMP response element binding (CREB) and activating transcription factor 2 (ATF-2). Accordingly, in that study, it significantly reduced the production of IL1β, tumor necrosis factor (TNF)α, IL6, and granulocyte-macrophage colony stimulating factor (GM-CSF) [21].

We used two animal models to evaluate the *in vivo *analgesic or antiarthritic effects of piperine. We found that piperine (100 mg/kg) effectively improved the symptoms of arthritic diseases with an effect comparable to prednisolone, although at 20 mg/kg, piperine did not have a significant analgesic effect in the arthritic animal model.

One of the major drawbacks of the current study was the large amount of piperine administered. Though the effects of 100 mg/kg piperine were therapeutic, several other studies have shown *in vivo *effects with doses below 50 mg/kg [22-24]. Furthermore, in this study, the effects reached significance at 8 or 9 days. Thus, the potency of piperine was relatively weak compared to 10 mg/kg prednisolone, which showed significant effects at 4 or 5 days. The *in vivo *toxicity of a 100 mg/kg dose piperine has not been tested; however, rats did not exhibit any adverse effects and they survived throughout the experiments. Nevertheless, piperine was shown to have immunotoxicological effects in mice at a dose of 2.25 mg/kg [25].

Piperine is also known to enhance the bioavailability of some drugs by inhibiting drug metabolism or by increasing absorption [18,24,26]. Thus, piperine may prove to be useful on combination treatments with other drugs. For example, a combination of gallic acid and piperine reduced beryllium-induced hepatorenal dysfunction and the associated oxidative stress [22]. In addition, a synergistic effect of piperine was demonstrated in a clinical study that tested the pharmacokinetics of nevirapine, a potent non-nucleoside inhibitor of HIV-1 reverse transcriptase [27]. The combination therapy was well tolerated, with few or no clinical adverse effects, and the mean maximum plasma concentration of nevirapine was increased when combined with piperine. In another clinical study, piperine was shown to increase the plasma levels of coenzyme Q10 [28]. Therefore, piperine may improve the therapeutic effect or lower the dose requirements of other drugs when administrated with DMARDs as a therapeutic drug or dietary supplement. In addition, combinations of DMARDs with piperine may reduce the side effects of DMARDs.

## Conclusions

For the first time, we have demonstrated that piperine has antirheumatic effects in animal models and anti-inflammatory effects on IL1β-stimulated FLSs. Our results suggest that piperine has potential as a therapeutic drug or dietary supplement. Thus, further investigations should focus on the development of piperine analogues that have potent efficacy and few adverse effects.

## Abbreviations

ELISA: enzyme-linked immunosorbent assay; FLS: fibroblast-like synoviocytes; H&E: hematoxylin and eosin; IL: interleukin; MMP: matrix metalloproteinase; OA: osteoarthritis; RA: rheumatoid arthritis; WDR: weight distribution ratio.

## Competing interests

The authors declare that they have no competing interests.

## Authors' contributions

KSK and DHH participated in the data analysis and the design of the study, and drafted the manuscript. JSB, DHO, HMC, BJS, JYK, and SJL performed the experiments. MCY and HIY provided the synovium from patients and participated in the design of the study. All authors read and approved the final manuscript.
